# Effect of Performance-Based Nonfinancial Incentives on Data Quality in Individual Medical Records of Institutional Births: Quasi-Experimental Study

**DOI:** 10.2196/54278

**Published:** 2024-04-05

**Authors:** Biniam Kefiyalew Taye, Lemma Derseh Gezie, Asmamaw Atnafu, Shegaw Anagaw Mengiste, Jens Kaasbøll, Monika Knudsen Gullslett, Binyam Tilahun

**Affiliations:** 1 Department of Health Informatics, Institute of Public Health, College of Medicine and Health Sciences, University of Gondar Gondar Ethiopia; 2 Ministry of Health, The Federal Democratic Republic of Ethiopia Addis Ababa Ethiopia; 3 Department of Epidemiology and Biostatistics, Institute of Public Health, College of Medicine and Health Sciences, University of Gondar Gondar Ethiopia; 4 Department of Health System and Policy, Institute of Public Health, College of Medicine and Health Sciences, University of Gondar Gondar Ethiopia; 5 Management Information Systems, University of South-Eastern Norway Drammen Norway; 6 Department of Informatics, University of Oslo Oslo Norway; 7 Faculty of Health & Social Sciences, Science Center Health & Technology, University of South-Eastern Norway Notodden Norway

**Keywords:** individual medical records, data quality, completeness, consistency, nonfinancial incentives, institutional birth, health care quality, quasi-experimental design, Ethiopia

## Abstract

**Background:**

Despite the potential of routine health information systems in tackling persistent maternal deaths stemming from poor service quality at health facilities during and around childbirth, research has demonstrated their suboptimal performance, evident from the incomplete and inaccurate data unfit for practical use. There is a consensus that nonfinancial incentives can enhance health care providers’ commitment toward achieving the desired health care quality. However, there is limited evidence regarding the effectiveness of nonfinancial incentives in improving the data quality of institutional birth services in Ethiopia.

**Objective:**

This study aimed to evaluate the effect of performance-based nonfinancial incentives on the completeness and consistency of data in the individual medical records of women who availed institutional birth services in northwest Ethiopia.

**Methods:**

We used a quasi-experimental design with a comparator group in the pre-post period, using a sample of 1969 women’s medical records. The study was conducted in the “Wegera” and “Tach-armacheho” districts, which served as the intervention and comparator districts, respectively. The intervention comprised a multicomponent nonfinancial incentive, including smartphones, flash disks, power banks, certificates, and scholarships. Personal records of women who gave birth within 6 months before (April to September 2020) and after (February to July 2021) the intervention were included. Three distinct women’s birth records were examined: the integrated card, integrated individual folder, and delivery register. The completeness of the data was determined by examining the presence of data elements, whereas the consistency check involved evaluating the agreement of data elements among women’s birth records. The average treatment effect on the treated (ATET), with 95% CIs, was computed using a difference-in-differences model.

**Results:**

In the intervention district, data completeness in women’s personal records was nearly 4 times higher (ATET 3.8, 95% CI 2.2-5.5; *P*=.02), and consistency was approximately 12 times more likely (ATET 11.6, 95% CI 4.18-19; *P*=.03) than in the comparator district.

**Conclusions:**

This study indicates that performance-based nonfinancial incentives enhance data quality in the personal records of institutional births. Health care planners can adapt these incentives to improve the data quality of comparable medical records, particularly pregnancy-related data within health care facilities. Future research is needed to assess the effectiveness of nonfinancial incentives across diverse contexts to support successful scale-up.

## Introduction

### Background

Maternal mortality, a pressing global health concern, is particularly prevalent in low- and middle-income countries [1-5]. The existing research attributes the persistence of maternal deaths largely to inadequate health care quality during labor, delivery, and immediate postpartum care in health facilities [6,7]. Almost every low- and middle-income country implements the Routine Health Information System (RHIS) to address this challenge [8-10]. The RHIS has gained prominence for its practical roles in improving the quality of services, including (1) facilitating evidence-based action by enabling the early detection of pregnancy-related complications, (2) serving as a repository for clients’ data to ensure the continuity of pregnancy-related care, and (3) functioning as the primary data source essential for health monitoring and evaluation at all levels of the public health system [11-16]. Despite its potential, the performance of RHIS remains suboptimal, primarily because of incomplete and inaccurate data, hindering its effective use by decision makers [17-20].

In Ethiopia, the introduction of the RHIS dates back to 2008 [21,22]. Ongoing efforts are in place to enhance the data quality of the RHIS in Ethiopia through interventions such as the Performance Monitoring Team (PMT), lot quality assurance sampling (LQAS), and the Capacity Building and Mentorship Program (CBMP) [23-25]. However, despite these efforts, the quality of RHIS data still lags in Ethiopia [15,26]. This challenge is pertinent to institutional birth, as shown by some previous studies in Ethiopia. For instance, a study [27] reported a completeness rate of only 18.4%. Another study from Ethiopia found that 66% of health facilities managed to produce accurate data within an acceptable range [28]. Furthermore, comparing the data from health facilities with external sources such as the Ethiopian Demographic Health Survey reveals concerns regarding data quality in Ethiopian RHIS [26].

### Incentives and Its Impact on Health Care Quality

Previous studies have shown that offering incentives for personnel responsible for data collection and management can improve data quality in the RHIS. According to some studies, incentives are essential for addressing the negative attitudes and values that undermine data quality within the RHIS, which are primary challenges to achieving desired quality of RHIS data [29].

The effectiveness of incentives in health care is grounded in theoretical and empirical evidence. Theories like the theory of planned behavior emphasize the connection between motivation and improved health care quality [30]. Some studies demonstrated that incentive-based interventions can predict up to 48% of desired health care behavior [31,32].

Despite the growing interest in using incentives in health care [32-38], determining the most effective approach remains a research priority. Incentives can be financial [39,40] or nonfinancial. Financial incentives have been extensively studied globally [33,37-39,41-43], but there is limited evidence supporting their consistent impact on health care quality [40]. Some studies have even cited the counterproductive effects of financial incentives on health care [44].

Compared to financial incentives, the impact of nonfinancial incentives on health care quality has been minimally studied [45]. Nonfinancial incentive schemes offer noncash rewards or benefits to motivate recipients using approaches that involve recognition through public profiling or reporting; career advancement opportunities; providing certificates to top performers; and ensuring improved working conditions, such as vacations, grading systems, and packaging interventions with in-kind items [46-53]. Previous studies have demonstrated the effectiveness of nonfinancial incentives in enhancing the quality of health services. For instance, nonfinancial incentive schemes in the United States, India, El Salvador, and Tanzania have been reported to enhance the performance of health care providers, including enhanced root cause data analysis of medical errors, expanded community outreach services, better maternal and child care services, and higher quality health care consultations [50,51,54,55].

### Objectives

This study aimed to evaluate the effect of performance-based nonfinancial incentives (PBNI) on enhancing the quality of institutional birth data, measured by the completeness and consistency of data within women’s individual medical records (IMRs).

## Methods

### Study Design and Period

This study used a quasi-experimental design with a comparator group in the pre-post period to examine the effect of PBNI on the data quality of IMRs of institutional births. A cross-sectional survey within an institutional setting was used to review institutional birth–related medical records. The study included the IMRs of women who gave birth within 6 months before (April to September 2020) and after (February to July 2021) the intervention.

### Study Setting

**Figure 1:**
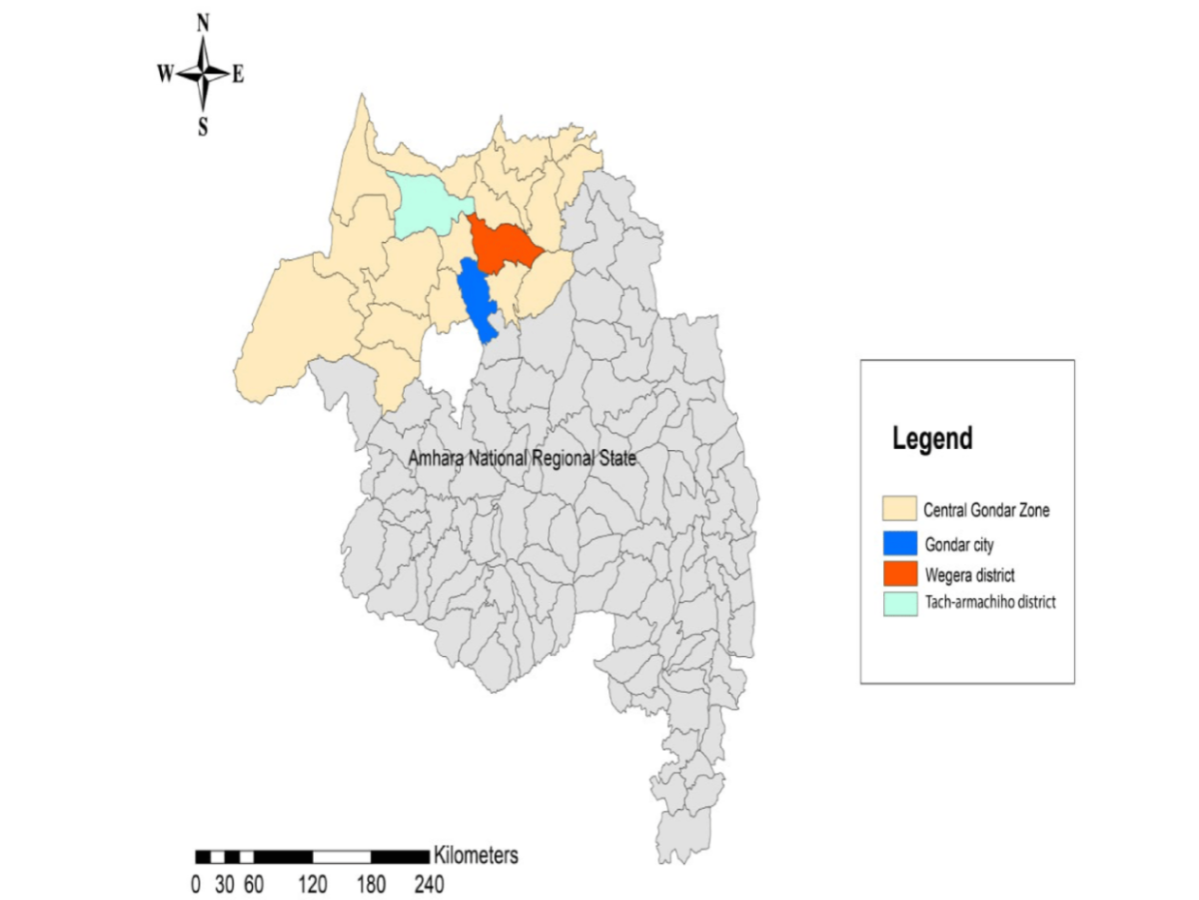
Map of the study area.

This study was conducted in the Amhara National Regional State, specifically in the Central Gondar Zone districts of “Wegera” and “Tach-armachiho” in northwest Ethiopia (Figure 1). According to the Ethiopian Central Statistical Agency projection, the total population in both districts was approximately 398,350 in 2021, with an estimated 93,214 women in the reproductive age group (15-49 years) [56,57]. The districts hosted 2 primary hospitals, 13 health centers, and 72 health posts. The intervention district had 678 health care providers and 215 support staff, whereas the comparator district had 202 health care providers and 141 support staff [58]. These districts were chosen for their involvement in the CBMP, a collaborative initiative between the Ethiopian Ministry of Health and the University of Gondar [24,59,60]. As part of the CBMP, the University of Gondar provides ongoing technical support, including training, supervision, and mentorship to health facilities in both districts, to enhance data quality and information use [59]. This study evaluated the effect of PBNI in the “Wegera” district, with the “Tach-armachiho” district serving as a comparator.

### Intervention

#### Intervention Aim and Design

The PBNI intervention was implemented in the “Wegera” district between October 2020 and July 2021 to improve the data quality and use of the RHIS. PBNI was designed as a package with multiple nonfinancial incentive components. Incentives were offered across 3 levels: health facilities, departments, and individual health care workers. Individual health workers were offered nonfinancial incentives, including smartphones, flash disks, power banks, and scholarships. Desktop computers were offered at the department and health facility levels. Health workers, departments, and health facilities that earned nonfinancial incentives in each round were awarded certificates of recognition [61].

#### Target Areas for PBNI

The study included 6 (75%) out of the 8 health centers in “Wegera.” The departments involved in the PBNI program include Maternal and Child Health, Outpatient Department, under-5 Outpatient Department, the Health Management Information System (HMIS), and the Medical Record Unit (MRU). Health workers who participated in the PBNI program included various experts, such as medical record personnel, health IT (HIT) personnel, health officers, midwives, nurses, and personnel involved in laboratories and pharmacies.

#### Awardees Selection Procedures

The selection of the best performers was conducted through 2 approaches: a subjective and an objective approach.

##### Subjective Approach

The subjective approach involved requesting management authorities in the intervention district to nominate the best-performing employees. The subjective approach was chosen owing to practical constraints, as the quantitative measurement of all health workers’ performance was infeasible owing to limited resources. Accordingly, the number of potential awardees was reduced to a manageable level, allowing us to concentrate on objectively evaluating the candidates.

The subjective approach was conducted in 2 phases. In the initial phase, health office department managers in the intervention district nominated 12 individuals, selecting 2 from each of the 6 participating health centers. The second phase mirrored the first phase, except that the selection process took place at the level of each health center, where the heads of each health center were tasked with nominating the best performers. With 2 nominees selected by the heads of each health center, another 12 individuals were identified. Consequently, 24 individuals were identified using a subjective approach.

##### Objective Approach

Previous research indicates that effective health care incentives depend on rewarding specific performance [62,63]. In this study, for the purpose of incentivizing 3 entities—health centers, departments, and individual health workers—we used a flexible approach that used objective measures to identify the best performers. For health centers, 14 quantitative performance measures, each of which was established with specific targets and points to be earned, were used (Textbox 1). The allocation of point values and performance targets took priority for the RHIS activities, as defined by the Ethiopian Ministry of Health [22].

The performance of departments and the 24 individuals selected during the subjective phases was objectively evaluated, aligning the 14 quantitative performance measures with their relevant roles and job descriptions. Further details on the performance measures used are described in prior studies [58,61,64,65].

**The performance indicators used to determine the awardees of nonfinancial incentives, northwest Ethiopia, 2021.**

**Indicators and points (total points: 90)**

1. Source documents completeness rate: 10
2. Report timeliness: 5
3. Lot quality assurance sampling performance: 6
4. Data consistency among registers and reports: 12
5. Health centers established by Performance Monitoring Team: 8
6. Action plan implemented regularly: 10
7. Conducted internal supervision: 5
8. Gaps identified and prioritized by Performance Monitoring Team: 5
9. Conducted root cause analysis: 5
10. Feedback provided for case teams by health centers: 4
11. Number of feedback entries provided to health posts by health centers 5:
12. Information display status: 5
13. Report completeness: 5
14. Consistency among medical records: 5

#### The Awarding Processes

Initially, the team from the University of Gondar visited the health office department and health centers in the intervention district to communicate the commencement of the program. During this announcement phase, a banner illustrating the nonfinancial rewards was displayed within the compounds of the health facilities. Nonfinancial incentives were offered to the recipients through 3 ceremonial award programs that took place bimonthly. The attendees of the PBNI ceremonial award include representatives from the University of Gondar, Federal Ministry of Health, Amhara Regional Health Bureau, Central Gondar Zone, and “Wegera” District Health Office departments. These representatives comprised health experts and administrative personnel. Officials from the Federal Ministry of Health and University of Gondar rewarded the top-3 individuals, departments, and health centers. Certificates of recognition were presented to awardees during these bimonthly forums. Ceremonial events were also accompanied by presentations detailing the performance measurement and award selection procedures by the professionals from the University of Gondar.

### Study Participants

#### Overview

The participants in this study were women who had given birth in the health centers at the study sites. The IMR sets of these individuals were examined. Thus, for each woman, there would be a set of 3 types of records: delivery register, integrated individual folder (IIF), and integrated card. These 3 sets of IMRs were combined to form a single study cohort. The 3 types of IMRs evaluated in this study, designed to record institutional birth data following the guidelines established in Ethiopia [66], are described in the following sections.

#### Integrated Card

The integrated card captures data on pregnant women throughout antenatal, labor, delivery, and postnatal care. It facilitates the recording of medical histories, physical examination results, and other clinical data for both women and newborns, allowing health care providers to complete it upon birth.

#### Delivery Register

The delivery register is a serial-long register designed to contain a list of all women who give birth at the facility, with data abstracted from the integrated card.

#### Women’s IIF

The IIF is designed to consolidate the entirety of a woman’s personal records, including the integrated card. It ensures the convenient access to comprehensive medical data, with the front section containing personal identification data filled out during registration and the inner part featuring a summary sheet completed by service providers after each visit.

### Sample Size Calculation

The required sample size for this study was calculated using StatCalc (Epi Info version 7.0; Centers for Disease Control and Prevention), incorporating assumptions to detect differences in completeness and consistency rates between the intervention and comparator groups. The assumptions included 80.3% completeness and 29.5% consistency from a prior unpublished pilot study (Taye, BK, unpublished data, September 2021), a 1:1 ratio of the intervention to the comparator group, a 5% anticipated change in the intervention group [55], 80% power, 95% CI, and a 10% nonresponse rate. Separate calculations yielded approximately 1969 participants (985 in each group) for completeness and approximately 3007 (1504 in each group) for consistency. From the 2 computed samples, we chose 1969 participants, considering the available resources for feasibility.

### Inclusion and Exclusion Criteria

A list of women who gave birth during the study period was used as a sampling frame to select the study sample. Three distinct IMRs in combination (delivery register, IIF, and integrated card) were necessary for each participant. The inclusion criteria in this study mandated that women’s “medical record numbers” (MRNs) and the mother’s full name be legibly recorded on the delivery register. The readability of these 2 variables was necessary to match and retrieve women’s IIF data from the MRU.

### Sampling Procedure

A total of 11 health centers were included in the pre- and postintervention periods, of which 6 (55%) were in the intervention district and 5 (45%) were in the comparator district. In total, 2426 women’s entries were identified from the delivery register across the baseline and end line periods.

**Figure 2:**
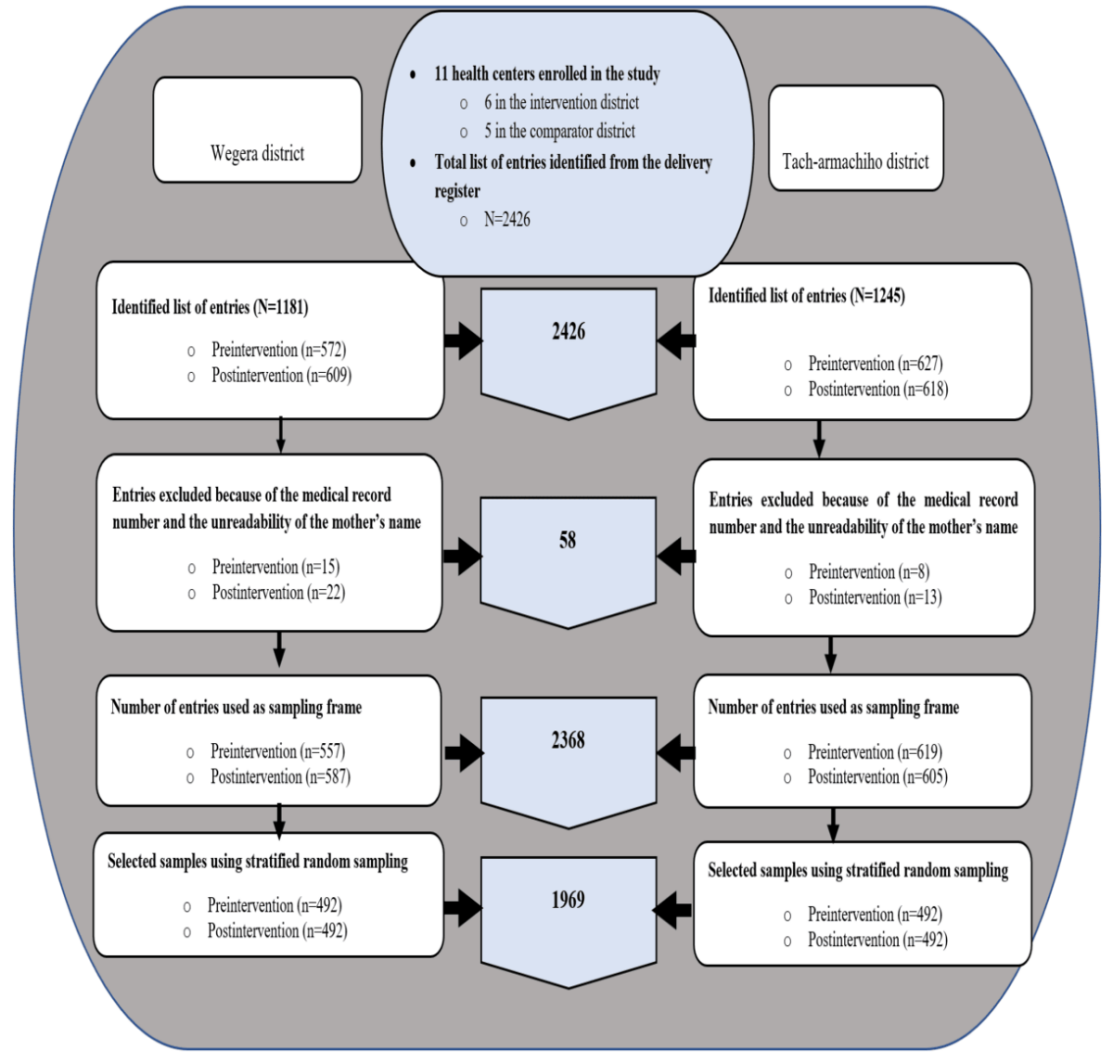
A diagram illustrating the process of study sample selection.

Of the 2426 entries, we excluded 58 (2%) due to unreadability of the MRNs and the full names of the mothers, resulting in 2368 (98%) entries of women on the delivery register. The list of 2368 women in the delivery register was used as a sampling frame to select 1969 (83.1%) samples through stratified random sampling. Subsequently, the women’s IIF was retrieved from the MRU (Figure 2).

### Variables and Measurements

#### Outcome Variables

##### Completeness

Completeness refers to the presence of data elements in the targeted data set [67]. Within the RHIS, assessing the completeness of data elements mandates the presence of its corresponding services or medical procedures in health facilities [68]. Accordingly, this research evaluated the completeness of the 33 data elements where the guideline necessitates their recording [66]. Of the 33 data elements, 16 (48%) were from the integrated card (mother’s name, gravida, para, MRN, date and time of admission, ruptured membranes, date and time of delivery, mode of delivery, placenta details, newborn’s sex, newborn outcome, single or multiple births, term or preterm status, and the name and signature of providers), 7 (21%) were from the individual folder (facility name, registration date, client’s sex, mother’s age, date of delivery, Department-provided service, and serial number), and 10 (30%) were from the delivery register (serial number, age, address, date and time of delivery, newborn outcome, mode of delivery, maternal status, newborn’s sex, and the name and signature of providers).

##### Consistency

According to previous research [69], consistency is a measure of data accuracy (the extent to which data elements accurately represent the true numbers), commonly assessed in RHIS through data verification (agreement of data among data sources). This research crosschecked the agreement between the data elements in the source documents to assess consistency. The delivery register served as the gold standard, allowing a comparison with 12 data elements abstracted from the IIF and integrated card, including the serial number, MRN, mother’s name, delivery date and time, mode of delivery, newborn’s sex, provider’s name and signature, Apgar score, newborn’s weight, mother’s HIV test acceptance, and mother’s HIV test results.

In this study, data collectors judged the completeness and consistency of data elements, and the decision adhered to the guidelines [66]. For completeness, data collectors observed the presence of data elements in personal records and declared and coded each data element as 1 for “Yes” if recorded and 0 for “No” if unrecorded. Regarding consistency, data collectors verified elements from comparable records for agreement, coding them as 1 for “Yes” if consistently recorded and 0 for “No” otherwise.

#### Independent Variables

The characteristics of health facilities supposed to be associated with the data quality of institutional birth were included in the analysis, drawing from pertinent literature and guidelines in the field [22,66,70-72]. These independent variables were the presence of HIT personnel, availability of data recording tools, availability of trained providers, supportive supervision from higher officials, existence of PMT, PMT per membership standard, monthly PMT meetings, monthly conducted LQAS, conducted root cause analysis (RCA) on the identified gaps, internal supervision, and availability of HMIS guidelines. Details of the measurement and data management for outcomes and independent variables have been outlined in a previous study [73].

### Data Collection

In total, 11 data collectors, HIT personnel, and related health sciences graduates were recruited for the data collection. The data collectors received a 3-day training that covered the study’s objectives, methodology, and ethical considerations. The principal investigator and the other 2 supervisors supervised the data collection process.

### Statistical Analysis

Data were entered into the EpiData (version 3.1; Epi Data Association) and exported into Stata (version17.0; StataCorp) for analysis.

#### Descriptive Statistics

The frequency distribution of women’s IMRs is presented based on the characteristics of the health facilities. Pearson chi-square tests were computed to assess the comparability of the data on the study participants’ baseline characteristics between the intervention and comparator groups.

The completeness and consistency proportions were computed for specific data elements, with the average rates calculated for the study participants. The completeness proportion of data elements was determined by dividing the number of participants with recorded data elements by the total number of study participants. Meanwhile, the mean completeness proportion was calculated by dividing the number of data elements recorded per participant by the total expected number of data elements. Likewise, the consistency proportion of data elements was determined by dividing the number of participants with consistently recorded data elements by the total number of study participants, and the mean consistency proportion was calculated by dividing the number of data elements consistently recorded per participant by the total expected number of data elements.

Changes in completeness and consistency proportions between the study groups were compared by computing absolute differences and their corresponding 95% CIs, using a 2-sample proportion test.

#### Difference-in-Differences Analysis

This study used difference-in-differences (DID) to estimate the average effect of PBNI on data completeness and consistency [74,75]. The average treatment effect on the treated (ATET) was computed using the DID model. The DID model used can be described as follows:



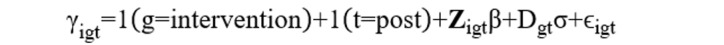

**(1)**


where γ*_i_* represents the outcome (either completeness or consistency) for each study participant. The variable 1(g = intervention) is an indicator for the study group, taking the value of “1” if the participant belongs to the intervention group and “0” otherwise. Similarly, the variable 1(t = post) is a binary indicator, taking the value of “1” for the participants sampled during the end line period and 0 otherwise. *Z_igt_β* represents the independent variables, that is, the characteristics of health facilities included in the DID model. *D* represents the intervention in this study (PBNI). σ represents the coefficient of average treatment effect on the intervention group (ATET), providing estimates of the average effect of the PBNI on the outcome variables, and 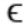
*_igt_* are the residual errors.

### Ethical Considerations

Ethics clearance was obtained from the University of Gondar’s Institutional Ethical Review Board (RNO: V/P/RCS/05/861-2021). As this study used medical records rather than human participants, obtaining informed consent from the participants was not feasible.

## Results

### Description of Women’s IMRs by Health Facilities’ Baseline Characteristics

In this study, 91.92% (1810/1969) of the samples met the analysis criteria for the coexistence of all 3 distinct IMRs. Of the analysis sample, 49.78% (901/1810) were sampled from the intervention district and 50.22% (909/1810) from the comparator district, whereas 49.56% (897/1810) were enrolled at baseline and 50.44% (913/1810) were enrolled at end line.

Among the baseline samples involved in the analysis, 51% (458/897) were enrolled from the intervention district and 48.9% (439/897) were from the comparator district. When comparing study participants according to baseline health facility characteristics, the distribution was identical (897/897, 100%) across the following variables: the presence of HIT personnel, the existence of PMT, the PMT established per membership standard, monthly PMT meetings in the last 6 months, and monthly LQAS in the previous 6 months.

Most of the IMRs (425/458, 92.8%) in the intervention district were from health centers where the data recording tools were fully available, compared with 12.3% (54/439) in the comparator district (*P*<.001). In the intervention district, 69% (316/458) of the IMRs were from health centers where most providers had received training on data quality, compared with 65.6% (288/439; *P*=.28) in the comparator district. In the intervention district, 39.7% (182/458) of the IMRs were from health centers with at least 3 supportive supervision visits by higher officials, compared with 65.6% (289/439; *P*<.001) in the comparator district. Nearly one-third of the IMRs (145/458, 31.6%) in the intervention district were from health centers that conducted at least 2 internal supervisions, compared with 4.8% (21/439; *P*<.001) in the comparator district. Less than three-fourth of the IMRs (303/458, 66.2%) in the intervention district were from health facilities that conducted RCA at least 3 times, compared with 25.2% (111/439; *P*<.001) in the comparator district. In the intervention district, 92.8% (425/458) of the IMRs were from health centers with fully available HMIS guidelines, compared with 91.8% (403/439; *P*=.58) in the comparator district (Table 1).

**Table 1:** Baseline comparison between the study groups by the characteristics of health facilities, northwest Ethiopia, 2021.

Variables			Intervention (N=458), n (%)		Comparator (N=439), n (%)		*P* value
Presence of health information technology personnel			458 (100)		439 (100)		<.001
Existence of PMT^a^			458 (100)		439 (100)		<.001
The PMT per membership standard			458 (100)		439 (100)		<.001
Monthly PMT meeting			458 (100)		439 (100)		<.001
Monthly conducted lot quality assurance sampling			458 (100)		439 (100)		<.001
**Availability of data recording tools**							
	Fully	425 (92.8)		54 (12.3)		<.001	
	Partially	33 (7.2)		385 (87.7)		<.001	
**Availability of trained providers**							
	Mostly	316 (69)		288 (65.6)		.28	
	Partially	142 (31)		151 (34.4)		.28	
**Supportive supervisions from higher officials**							
	<3 times	276 (60.2)		151 (34.4)		<.001	
	At least 3 times	182 (39.7)		289 (65.6)		<.001	
**Conducted** **root cause analysis** **on the identified gap**							
	Yes	303 (66.2)		111 (25.2)		<.001	
	No	155 (33.8)		328 (74.7)		<.001	
**Internal supervision**							
	At least 2 times	145 (31.6)		21 (4.8)		<.001	
	<2 times	313 (68.3)		418 (95.2)		<.001	
**Availability of the Health Management Information System guidelines**							
	Fully available	425 (92.8)		403 (91.8)		.58	
	Partially available	33 (7.2)		36 (8.2)		.58	

^a^PMT: Performance Monitoring Team.

### Specific Data Elements Completeness Across Study Groups

Table 2 compares the intervention and comparator districts regarding the completeness of specific data elements across the 3 IMRs. Concerning the data elements from the integrated card, the “Name of the mother” showed 95.6% (861/901) completeness in the intervention district compared with 92.6% (842/909; *P*=.004) in the comparator district. The completeness proportion of “Gravida” was 91.7% (826/901) in the intervention district, compared with 90.4% (822/909; *P*=.18) in the comparator district. The completeness proportion of “MRN” was 92.7% (835/901) in the intervention district, compared with 84.9% (771/909; *P*<.001) in the comparator district. The completeness proportion of “Time of Admission” was 92.5% (833/901) in the intervention district, compared with 88.7% (806/909; *P*=.003) in the comparator district. The completeness proportion of “time of delivery” was 94.9% (855/901) in the intervention district, compared with 89.6% (814/909; *P<*.001) in the comparator district. The completeness proportion of “Name and signature of providers” was 90.1% (812/901) in the intervention district, compared with 86.8% (789/909; *P*=.01) in the comparator district.

In the IIF, the “date of delivery” completeness proportion was 39.3% (354/901) in the intervention district, compared with 29.4% (268/909; *P*<.001) in the comparator district. The completeness proportion of “Date of registration” was 99.8% (900/901) in the intervention district, compared with 93.4% (849/909; *P*<.001) in the comparator district. The completeness proportion of “Department-provided service” was 37.1% (334/901) in the intervention district, compared with 29.4% (267/909; *P*<.001) in the comparator district. The completeness proportion of “Serial Number” was 35.2% (318/901) in the intervention district, compared with 31% (282/909; *P*=.03) in the comparator district.

Regarding the data elements in the delivery register, the “Serial Number” and “Age” were found to be recorded for all study participants across the intervention and comparator districts. In the intervention district, the “time of delivery” showed 92.7% (835/901) completeness, compared with the comparator district, which showed 62.6% (571/909; *P*<.001) completeness. In the intervention district, the “date of delivery” showed 99.7% (899/901) completeness, compared with the comparator district, which showed 95.3% (867/909; *P*<.001) completeness. The completeness proportion of “sex of newborn” was 99.4% (896/901) in the intervention district, compared with 99% (900/909; *P*=.14) in the comparator district. The “Name and signature of providers” completeness proportion was 78.3% (706/901) in the intervention district, compared with 80.9% (736/909; *P*=.92) in the comparator district.

**Table 2:** Specific data elements’ completeness in individual medical records of institutional births across intervention and comparator districts, northwest Ethiopia, 2021.

Completeness		Intervention (N=901), n (%)	Comparator (N=909), n (%)	Difference^a^ (95% CI)	*P* value^b^
**Integrated card**					
	Name of the mother	861 (95.6)	842 (92.6)	2.9 (0.76 to 5)	.004
	Gravida	826 (91.7)	822 (90.4)	1.2 (−1.48 to 3.8)	.18
	Para^c^	858 (95.2)	820 (90.2)	5 (2.6 to 7.49)	*<*.001
	Medical record number	835 (92.7)	772 (84.9)	7.7 (4.8 to 10.6)	*<*.001
	Date of admission	841 (93.3)	822 (90.4)	2.9 (0.4 to 5.4)	.01
	Time of admission	833 (92.5)	806 (88.7)	3.8 (1 to 6.4)	.003
	Ruptured membranes	664 (73.7)	760 (83.6)	9.9 (−13.6 to −6.26)	>.99
	Date of delivery	861 (95.6)	843 (92.7)	2.8 (0.6 to 4.9)	.005
	Time of delivery	855 (94.9)	814 (89.6)	5.3 (2.8 to 7.89)	*<*.001
	Mode of delivery	834 (92.6)	806 (88.7)	3.8 (1.2 to 6.6)	.002
	Placenta	842 (93.5)	804 (88.5)	5 (2.4 to 7.6)	*<*.001
	Sex of the newborn	851 (94.5)	813 (89.4)	5 (2.5 to 7.5)	*<*.001
	Newborn outcome	850 (94.3)	822 (90.4)	3.9 (1.5 to 6.3)	*<*.001
	Single or multiple	756 (83.9)	802 (88.2)	4.3 (−7.5 to −1.1)	>.99
	Term or preterm	786 (87.2)	801 (88.1)	0.8 (−3.9 to 2.1)	.72
	Name and signature	812 (90.1)	789 (86.8)	3.3 (0.38 to 6.26)	.01
**Integrated individual folder**					
	Name of the facility	887 (98.4)	807 (88.7)	9.6 (7.4 to 11.8)	*<*.001
	Date of registration	900 (99.8)	849 (93.3)	6.4 (4.8 to 8.1)	*<*.001
	Sex of the client	899 (99.7)	858 (94.3)	5.4 (3.8 to 6.9)	*<*.001
	Age of the mother	843 (93.5)	860 (94.6)	1 (−3.2 to 1.1)	.83
	Date of delivery	354 (39.2)	268 (29.4)	9.8 (5.4 to 14.2)	*<*.001
	Department-provided service	334 (37)	267 (29.3)	7.6 (3.4 to 12)	*<*.001
	Serial number	318 (35.2)	282 (31)	4.3 (−0.01 to 8.6)	.03
**Delivery register**					
	Serial number	901 (100)	909 (100)	—^d^	*<*.001
	Age	901 (100)	909 (100)	—	*<*.001
	Address	900 (99.8)	900 (99)	0.8 (0.2 to 1.6)	.005
	Date of delivery	899 (99.7)	867 (95.3)	4.4 (2.9 to 5.8)	*<*.001
	Time of delivery	835 (92.6)	571 (62.8)	29.8 (26.2 to 33.4)	*<*.001
	Newborn outcome	897 (99.5)	905 (99.5)	—	*<*.001
	Mode of delivery	898 (99.6)	899 (98.8)	0.8 (−0.01 to 1.5)	.05
	Maternal status	897 (99.6)	909 (100)	0.4 (−0.8 to –0.01)	.98
	Sex of the newborn	896 (99.4)	900 (99)	0.4 (−0.37 to 1.24)	.14
	Name and signature	706 (78.3)	736 (80.9)	2.6 (−6.31 to 1.09)	.92

^a^The absolute difference is calculated by subtracting the completeness proportion of the comparator group from that of the intervention group.

^b^*P* value based on 2 independent sample proportion tests.

^c^A number of times a woman has given birth to a viable child.

^d^No difference among intervention and comparator group.

### Average Data Completeness Across the Pre- and Postintervention Periods

For the integrated card, the average completeness increased from 86.2% (95% CI 83.9%-88.57%) at the baseline to 96.6% (95% CI 96%-97.1%) at the end line in the intervention district; however, in the comparator district, it showed a decrease from 91.1% (95% CI 89.4%-92.7%) at the baseline to 87% (95% CI 84.2%-89.7%) at the end line.

The average completeness of the IIF increased from 58.9% (95% CI 57.6%-60.2%) at the baseline to 85.3% (95% CI 83.6%-87%) at the end line in the intervention district, whereas the comparator district showed a change from 63.5% (95% CI 61.5%-65.4%) to 68.1% (95% CI 65.6%-70.6%).

In the intervention district, the mean completeness proportion of the delivery register increased from 94.6% (95% CI 93.9%-95.2%) at the baseline to 99.3% (95% CI 99%-99.5%) at the end line. In comparison, the comparator district showed a change from 93.5% (95% CI 92.9%-94%) to 93.6% (95% CI 92.9%-94.3%).

In the intervention district, the average data completeness proportion across the 3 individual IMRs was 82.9% (95% CI 81.88%-4.1%) at the baseline, and it increased to 95% (95% CI 94.6%-95.5%) at the end line. In the comparator district, the average data completeness proportion across the 3 IMRs was 86% (95% CI 84.96%-86.97%) at the baseline but decreased to 84.97% (95% CI 83.28%-86.66%) at the end line (Multimedia Appendix 1).

### Effect of PBNI on Data Completeness

In the intervention district, the “integrated card” resulted in an average 2.6 percentage-point increase in completeness compared with the comparator district (ATET 2.67, 95% CI 0.7-4.4; *P*=.04). On average, the intervention district showed a 3.8 percentage-point increase in the completeness of the delivery register compared with the comparator district (ATET 3.8, 95% CI 2.9-4.8; *P=*.01). The intervention district showed a 6.8 percentage-point increase in the average completeness of the IIFs compared with the comparator district (ATET 6.8, 95% CI 4.55-9; *P*=.02). Overall, on average, the intervention district showed a 3.8 times higher chance of complete recording of IMRs compared with the comparator district (ATET 3.8, 95% CI 2.2-5.5; *P*=.02; Table 3).

**Table 3:** Effect of performance-based nonfinancial incentives on the data completeness in individual medical records of institutional births, northwest Ethiopia, 2021.

Completeness	Intervention, mean (SD)	Comparator, mean (SD)	Intervention effect, ATET^a^ (95% CI)^b^	*P* value
Integrated card	91.3 (18.8)	88.9 (24.9)	2.6 (0.7-4.4)	.04
Integrated individual folder	71.9 (21.1)	65.8 (24.8)	6.8 (4.6-9)	.02
Delivery register	96.8 (5.8)	93.6 (6.9)	3.8 (2.9-4.8)	.01
Overall	88.8 (11.2)	85.4 (15.3)	3.8 (2.2-5.5)	.02

^a^ATET: average treatment effect on the treated.

^b^ATET estimates adjusted for covariates (presence of health information technology personnel, availability of data recording tools, availability of trained providers, supportive supervision from higher officials, existence of the Performance Monitoring Team [PMT], PMT per membership standard, monthly PMT meeting, monthly conducted lot quality assurance sampling, conducted root cause analysis, internal supervision, and availability of Health Management Information System guidelines).

### Consistency of Specific Data Elements Across Study Groups

Regarding the delivery register and IIF, the “date of delivery” showed a consistency proportion of 82.3% (742/901) in the intervention district, compared with 58.7% (534/909; *P<*.001) in the comparator district. The “Serial Number” showed a consistency proportion of 42.1% (380/901) in the intervention district, compared with 45.5% (414/909; *P*=.92) in the comparator district.

Comparing the delivery register and integrated card, the “MRN” exhibited a consistency proportion of 87% (784/901) in the intervention district, compared with 74.9% (681/909; *P<*.001) in the comparator district. The “time of delivery” showed a consistency proportion of 88.2% (795/901) in the intervention district, compared with 56.7% (516/909; **P*<*.001) in the comparator district. The “Name and signature of providers” showed a consistency proportion of 89.5% (807/901) in the intervention district, compared with 77.7% (707/909; *P<*.001) in the comparator district. The “newborn weight” had a consistency proportion of 85.7% (773/901) in the intervention district, compared with 82.1% (746/909; *P*=.01) in the comparator district. The “Women’s HIV test accepted” showed a consistency proportion of 39.9% (360/901) in the intervention district and 40.5% (368/909; *P*=.59) in the comparator district (Table 4).

**Table 4:** Consistency of specific data elements across the intervention and comparator districts, northwest Ethiopia, 2021.

Consistency		Intervention (N=901), n (%)	Comparator (N=909), n (%)	Difference^a^ (95% CI)	*P* value^b^
**Delivery register vs integrated individual folder**					
	Date of delivery	742 (82.3)	534 (58.7)	23.6 (19.55 to 27.66)	<.001
	Serial number	380 (42.1)	414 (45.5)	3.36 (−7.93 to 1.2)	.92
**Delivery register vs integrated card**					
	Medical record number	784 (87)	681 (74.9)	12.09 (8.52 to 15.66)	<.001
	Name of the mother	828 (91.8)	709 (77.9)	13.90 (10.67 to 17.12)	<.001
	Date of delivery	859 (95.3)	803 (88.3)	6.99 (4.50 to 9.49)	<.001
	Time of delivery	795 (88.2)	516 (56.7)	31.46 (27.62 to 35.31)	<.001
	Mode of delivery	831 (92.2)	796 (87.5)	4.66 (1.89 to 7.42)	<.001
	Sex of the newborn	846 (93.9)	806 (88.6)	5.22 (2.64 to 7.81)	<.001
	Name and signature	807 (89.5)	707 (77.7)	11.78 (8.42 to 15.14)	<.001
	Apgar score	781 (86.6)	746 (82.1)	4.61 (1.27 to 7.95)	.003
	Newborn weight	773 (85.7)	746 (82.1)	3.72 (0.34 to 7.1)	.01
	Women’s HIV test accepted	360 (39.9)	368 (40.4)	0.52 (−5.04 to 3.98)	.59
	Women’s HIV test result	615 (68.2)	381 (41.9)	26.34 (21.92 to 30.76)	<.001

^a^The absolute difference is calculated by subtracting the consistency proportion of the comparator group from that of the intervention group.

^b^*P* value based on 2 independent sample proportion tests.

### Pre- and Postintervention Changes in Average Data Consistency

In the intervention district, the average consistency proportion increased from 71.6% (95% CI 69.6%-73.6%) to 89.2% (95% CI 88.2%-90.2%) after the intervention. In the comparator district, it increased from 68% (95% CI 66.2%-69.8%) to 70.8% (95% CI 67.9%-73.6%) post intervention. Overall, the average consistency proportion increased from 69.8% (95% CI 68.5%-71.2%) to 79.6% (95% CI 78%-81.3%) after the intervention.

### Effect of PBNI on Data Consistency

On average, the intervention district showed an 11.2 percentage-point increase in the consistency of data among the delivery register and IIF compared with the comparator district (ATET 11.2; 95% CI 9.6- 12.87; *P*=.007). The intervention district showed an 11.6 percentage-point increase in the average consistency of data among the delivery register and the integrated card compared with the comparator district (ATET 11.6; 95% CI 3.1-20.1; *P*=.04). Overall, the average consistency of data among IMRs in the intervention district was 11.6 times higher than that of the comparator district (ATET 11.6; 95% CI 4.2- 19; *P*=.03; Table 5).

**Table 5:** Effect of performance-based nonfinancial incentives on data consistency in individual medical records of institutional births, northwest Ethiopia, 2021.

Consistency	Intervention, mean (SD)	Comparator, mean (SD)	Intervention effect, ATET^a^ (95% CI)^b^	*P* value
Delivery register vs integrated individual folder	62.2 (36.4)	51.1 (45.5)	11.2 (9.6-12.8)	.007
Delivery register vs integrated card	83.5 (19.9)	72.6 (26.0)	11.6 (3.1-20.1)	.04
Overall	80.2 (19.1)	69.4 (26.1)	11.6 (4.2-19)	.03

^a^ATET: average treatment effect on the treated.

^b^ATET estimates adjusted for covariates (presence of health information technology personnel, availability of data recording tools, availability of trained providers, supportive supervision from higher officials, existence of the Performance Monitoring Team [PMT], PMT per membership standard, monthly PMT meeting, monthly conducted lot quality assurance sampling, conducted root cause analysis, internal supervision, and availability of Health Management Information System guidelines).

## Discussion

### Principal Findings

This study evaluated the effect of PBNI on the quality of institutional birth data in northwest Ethiopia. PBNI improved both data completeness and consistency. The intervention district showed a 12% increase in data completeness compared with the comparator district, which showed a 1% decrease. Regarding data consistency, the intervention district improved by 18%, whereas the comparator district saw a 3% improvement. Controlling for other variables in the DID analysis, women’s IMRs from the intervention district exhibited nearly 4 times higher data completeness and approximately 12 times greater data consistency than the comparator district.

### Comparison With Prior Work

This study revealed a positive effect of PBNI on the data completeness and consistency of women’s IMRs for institutional births. This finding aligns with that of previous studies that demonstrate the effectiveness of nonfinancial incentives in improving different aspects of health care quality. For instance, nonfinancial incentives have been proven to enhance the quality of medical error RCA in a US study [50]. Furthermore, studies from India and El Salvador [51,54,76] have shown an increase in the equitable and quality provision of maternal and child services. Nonfinancial incentives have also been reported to enhance quality consultations, according to studies from Tanzania [55,77]. The demonstrated effectiveness across contexts suggests the adaptability of nonfinancial incentives to improve the data quality and the quality of pregnancy-related services at health care facilities. These findings are particularly relevant for resource-limited settings where poor health care quality is associated with persistent mortality rates among mothers and children [3,5,78].

In this study, PBNI induced a greater extent of change compared with that in previous studies [51,54]. This difference may be due to differences in the incentive structures among the studies. In contrast to prior research that used team-based incentives, this study provided incentives at 3 levels: health facilities, departments, and individual health workers. Notably, the similarity between previous and current studies is apparent in the use of team-based incentives, reflected in this study’s provision of incentives at the departmental level. Some earlier studies support the efficacy of team-based incentives in health care, emphasizing their role in fostering collective engagement [79-84]. Despite variations in the magnitude of the effect, the findings of this study do not contradict earlier research on the effectiveness of team-based incentives. Instead, the findings assert the potential for increased effectiveness by combining team-based, individual, and facility-level incentives.

According to this study, the effect of PBNI on data quality varies across the 3 women’s records (integrated card, IIF, and delivery register). For example, although the integrated card saw a 3% increase in data completeness, women’s folders increased by approximately 7%. These variations may suggest that the effectiveness of PBNI varies across health workers’ professions. In Ethiopia, for instance, nonprofessional health workers are largely responsible for women’s folder data, whereas midwives and other professionals are responsible for recording integrated card data [66]. Previous research in India has also demonstrated that the effectiveness of nonfinancial incentives varies depending on the health workers’ professions, with frontline health workers experiencing a greater performance than supervisors [54]. Another study in northwest Ethiopia found a strong correlation between health worker motivation and their professional category [85]. These findings indicate the importance of recognizing the differences in the effectiveness of incentives and tailoring interventions to specific groups of health workers. Hence, policy makers and health care managers need to consider these variations when designing incentive programs, adopting a flexible approach that accounts for diverse roles and responsibilities.

This study reinforces the existing evidence that favors nonfinancial incentives in health care over financial incentives [86-90]. Concerns about financial incentives contradicting health care providers’ intrinsic motivation to deliver quality care are widespread [86,90-100]. Therefore, this study suggests the practical use of nonfinancial incentives to enhance health care quality, especially in countries such as Ethiopia with limited financial capability. Prior studies in African countries have also indicated the importance of nonfinancial incentives in improving health care quality [44,101,102].

### Policy and Research Implications

This study introduces PBNI as an effective intervention to improve the quality of institutional birth data. These findings underscore the potential of PBNI to complement established interventions to enhance RHIS performance, such as supportive supervision, mentorship, training, and feedback.

As this study evaluated the effect of PBNI on institutional birth data—a core indicator of maternal health care quality—the implications extend to broader RHIS data related to maternal and child services. These findings indicate the potential of PBNI to improve the quality of health services, which can contribute to maternal and child morbidity and mortality reduction. Hence, health care planners can consider adapting PBNI to improve the quality of maternal and child health services.

This study examined the effectiveness of PBNI in the context of health workers in health centers. Future studies are essential to understand the impact of PBNI on health staff across diverse settings, including health posts and hospitals.

### Strengths and Limitations of the Study

One of the strengths of this study lies in its evaluation of the effect of PBNI on data quality within women’s IMR in institutional births. Unlike most previous studies on RHIS data quality, which studied health facilities as the unit of analysis, this study delved into the individual level of data quality, which is essential to understanding how PBNI influences client-level service quality. In addition, we attempted to detect the minimum effect of the PBNI, using a sufficient study sample, and compared the intervention with comparator sites, increasing the robustness of the findings. Furthermore, to establish a causal effect of PBNI, the study used DID analysis, a recognized causal analysis technique in nonrandomized studies. Nevertheless, it is essential to recognize the limitations of this study. First, the retrospective design prevented randomization in the selection of the study participants. Second, interviewer bias is possible during the completeness and consistency assessments, as data collectors judged these aspects despite training to reduce bias. Although we attempted to disentangle the effect of PBNI from other potential factors, unmeasured confounders may still exist. Moreover, the security issues in Northern Ethiopia might have disrupted the effectiveness of the PBNI, as the intervention coincided with security problems in the adjacent regions.

### Conclusions

This study shows that PBNI improves institutional birth data quality, as demonstrated by enhanced completeness and consistency. The effectiveness of PBNI can be extended to enhancing comparable RHIS data in maternal and child care and improving service quality at health care facilities. Health care planners can consider PBNI to enhance the quality of maternal and child health services in health care facilities. Future studies are essential to understand the impact of PBNI in diverse health care settings.

## Appendix

Multimedia Appendix 1 Average data completeness across individual medical records of institutional births during pre- and postintervention periods, northwest Ethiopia, 2021.

## Abbreviations

ATET: average treatment effect on the treated
CBMP: Capacity Building and Mentorship Program
DID: difference-in-differences
HIT: health IT
HMIS: Health Management Information System
IIF: integrated individual folder
IMR: individual medical record
LQAS: lot quality assurance sampling
MRN: medical record number
MRU: Medical Record Unit
PBNI: performance-based nonfinancial incentives
PMT: Performance Monitoring Team
RCA: root cause analysis
RHIS: Routine Health Information System
