# Supplementary figures and images for: Effect of Performance-Based Nonfinancial Incentives on Data Quality in Individual Medical Records of Institutional Births: Quasi-Experimental Study
Source: JMIR Med Inform. 2024 Apr 5;12:e54278. doi: 10.2196/54278 (PMC11031696; doi:10.2196/54278)

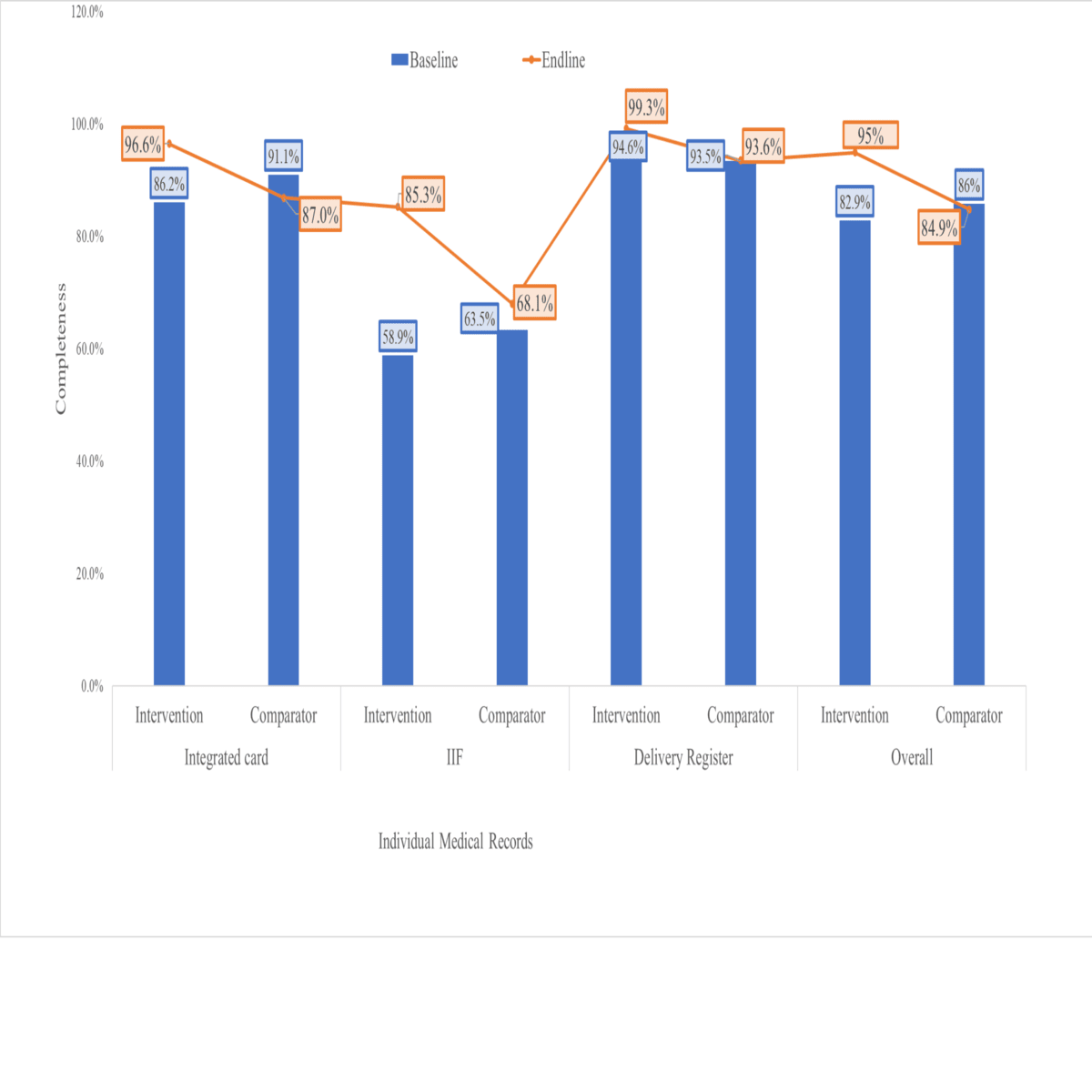

Supplement: Multimedia Appendix 1 [file medinform_v12i1e54278_app1.png]
